# One versus two anterior miniscrews for correcting upper incisor overbite and angulation: a retrospective comparative study

**DOI:** 10.1186/s40510-020-00336-2

**Published:** 2020-09-07

**Authors:** Arturo Vela-Hernández, Laura Gutiérrez-Zubeldia, Rocío López-García, Verónica García-Sanz, Vanessa Paredes-Gallardo, José Luis Gandía-Franco, Felicidad Lasagabaster-Latorre

**Affiliations:** 1 Orthodontics Teaching Unit, Department of Stomatology, University of Valencia, Gascó Oliag 1, 46010 Valencia, Spain; 2 In private practice, Vitoria, Spain

**Keywords:** Overbite, Incisor intrusion, Miniscrews, Resorption

## Abstract

**Background:**

Miniscrews are effective devices for performing upper incisor intrusion. Different mechanics can be applied depending on the treatment objectives. This study aimed to evaluate the efficacy of one or two anterior miniscrews for upper incisor correction in cases of overbite and angulation in adult patients.

**Methods:**

Forty-four adults with deep overbite were divided into two groups: *group 1* was treated with one miniscrew between upper central incisors and *group 2* with two miniscrews between upper lateral incisors and canines. Incisor intrusion and length were measured from lateral cephalograms before treatment, after treatment and at least 12 months into retention (T0, T1 and T2). Forces were applied (90 g) from the miniscrews to the archwire using elastomeric chains. ANOVA analysis was used to determine whether differences between evaluation times were statistically significant.

**Results:**

Mean root resorption was 2.15 ± 0.85 mm, which ceased after active treatment. Overbite mean correction was − 3.23 ± 1.73 mm with no statistically significant relapse. Overbite correction and incisor intrusion were significantly greater in *group 2* (− 3.80 ± 1.43 versus − 2.75 ± 1.63 for OB and 8.19 ± 3.66 versus 5.69 ± 2.66 for intrusion). Resorption and overbite correction were positively related. No counterclockwise rotation of the mandibular plane was observed.

**Conclusions:**

Overbite correction can be performed by means of upper incisor intrusion without rotation of the mandibular plane. Correction of upper incisor intrusion and overbite is greater in patients treated with two miniscrews. The increase in upper incisor buccal angulation is greater with one miniscrew. Root resorption is positively related to the extent of intrusion. Stability is satisfactory regardless of whether one or two miniscrews are used.

## Introduction

Vertical malocclusions with deep overbite can be treated with orthodontics alone or in combination with orthognathic surgery. The choice of one approach or the other will depend on the etiology and severity of the problem, as well as other individual factors such as the extent of gummy smile [1]. When surgical treatment is not an option because of the patient’s refusal to undergo surgery, or because no maxillary vertical excess is present, the use of miniscrews is a treatment option that offers an effective method for attaining maxillary incisor intrusion and correcting the gummy smile [2].

Miniscrews offer the advantages of immediate loading, a range of possible placement sites, relatively simple placement and removal, and low economic cost [3, 4]. Intrusion of upper and lower incisors, reducing overbite, can be easily achieved by placing miniscrews in anterior interradicular areas and applying the appropriate orthodontic mechanics. One or two miniscrews may be placed between central incisors [5, 6], central and lateral incisors [7], or lateral incisors and canines [8–12] and, providing the miniscrew (or screws) are located correctly [13], a good outcome with minimal incisor protrusion can be obtained.

Other auxiliary methods can be used to intrude upper incisors. Most of them use posterior teeth for anchorage, although this may produce unwanted reciprocal effects. An intrusion archwire is often used for overbite correction [14–17]. Comparing intrusion archwires with miniscrews, some authors have reported significantly more incisor proclination when using intrusion archwires [15], while others have found significantly more intrusion and generally better results using miniscrews [17].

Most of the studies quantifying upper incisor intrusion have used lateral cephalograms to perform measurements [7, 9, 12, 17, 18], while a few have evaluated root resorption using CBCT sagittal sections [14, 19].

Although the efficacy of anteriorly vs. posteriorly located miniscrew-assisted intrusion mechanics has been investigated, together with the resorptive root damage derived from miniscrew placement in different locations [19–22], no clinical trials have compared the effects (including root resorption) of treatment with 1 or 2 miniscrews placed in the anterior area. As both the forces applied and the vector position are different depending on whether one or two miniscrews are used, differences in the displacement pattern may occur, which could affect root resorption and treatment stability.

Therefore, the purpose of this study was to evaluate the results of orthodontic movement produced by one and two anterior miniscrews for upper incisor correction of overbite and angulation in adult patients.

## Materials and methods

This retrospective comparative human study was designed following STROBE guidelines and complied with the Helsinki Declaration for research involving human subjects. The study protocol was approved by the University of Valencia Ethics Committee for Human Research (Reg. No. 1069224). All patients whose records were used in the study received detailed information about its purpose and gave their informed consent to take part.

### Patients

Data from 90 patients attending a private dental clinic between January 2013 and December 2015 were used in the study; all these patients had been diagnosed with overbite and gummy smile.

Inclusion criteria were as follows:

- Non-growing patients. Lateral cephalograms of the patients were analyzed to assess skeletal growth using the cervical vertebral maturation method [23].
- Gummy smile of 3 mm or greater, diagnosed by examining the patient directly.
- Patients with incisor inclination smaller than 110° (U1-PP).
- Increased overbite diagnosed from lateral cephalograms by measuring the distance between upper and lower incisors’ incisal edges along a line perpendicular to the occlusal plane.
- Patients treated without extractions.
- Patients with good quality lateral cephalograms taken before treatment, just after treatment, and 12 or more months later during the retention period.
- Skeletal class I (ANB 2° ± 1).
- No periodontal surgery required in the incisor area as part of treatment.
- Patients treated with one or two anterior miniscrews.

Exclusion criteria were as follows:

-Patients with a history of any kind of trauma or endodontic treatment of the maxillary incisors.

-Patients presenting systemic disease or taking periodic medication.

-Patients exhibiting poor oral hygiene.

## Method

All patients were treated using fixed Tip-Edge Plus® (TP Orthodontics Inc.) bracket appliances (metallic or ceramic) and miniscrews in the upper anterior area. 0.014-in superelastic nickel-titanium (SE NT) archwires were applied to level and align maxillary and mandibular arches together with an upper 0.016-in A.J. Wilcock Australian stainless steel wire (G&H Orthodontics®_,_ Franklin, USA), followed by 0.016 × 0.025-in SE NT archwires to define the arch shape and level the occlusal plane. Stainless steel 0.021 × 0.028-in archwires combined with 0.016-in SE NT archwires, introduced through the auxiliary slot, were placed to perform correct torque and tipping. At this point, intermaxillary elastics were used if needed to make final occlusion adjustments. Finally, 0.016-in SE NT archwires were placed for optimal interdigitation. Lastly, appliances were removed and upper and lower canine-to-canine fixed lingual retainers were bonded. Upper and lower clear removable retainers were delivered to the patients to be used at night, adjusted to avoid anterior occlusal contact.

### Miniscrew mechanics

Miniscrews were placed in the upper incisor area to obtain intrusion of the upper incisors and to correct the gummy smile (length 8 mm; diameter 1.6 mm; head 2.3 mm, Dual Top, Jeil Medical Corporation, Seoul, South Korea) during the first treatment stage when brackets were bonded. The screws were inserted in the interradicular areas under local anesthesia, perpendicular to the teeth in order to endure the intrusion forces. Each miniscrew was used as a direct anchorage unit, applying a 90 g force after placing the 0.014-in SE NT and Australian stainless steel archwires. Australian wire was used at this point so that the intrusion forces applied from the miniscrews would be distributed more evenly between the six anterior teeth. Traction from the miniscrews was reactivated monthly. Intrusion forces from the miniscrews were applied until overbite correction was achieved. All miniscrews were placed by the same experienced operator (AVH) using a straight screwdriver.

**Fig. 1:**
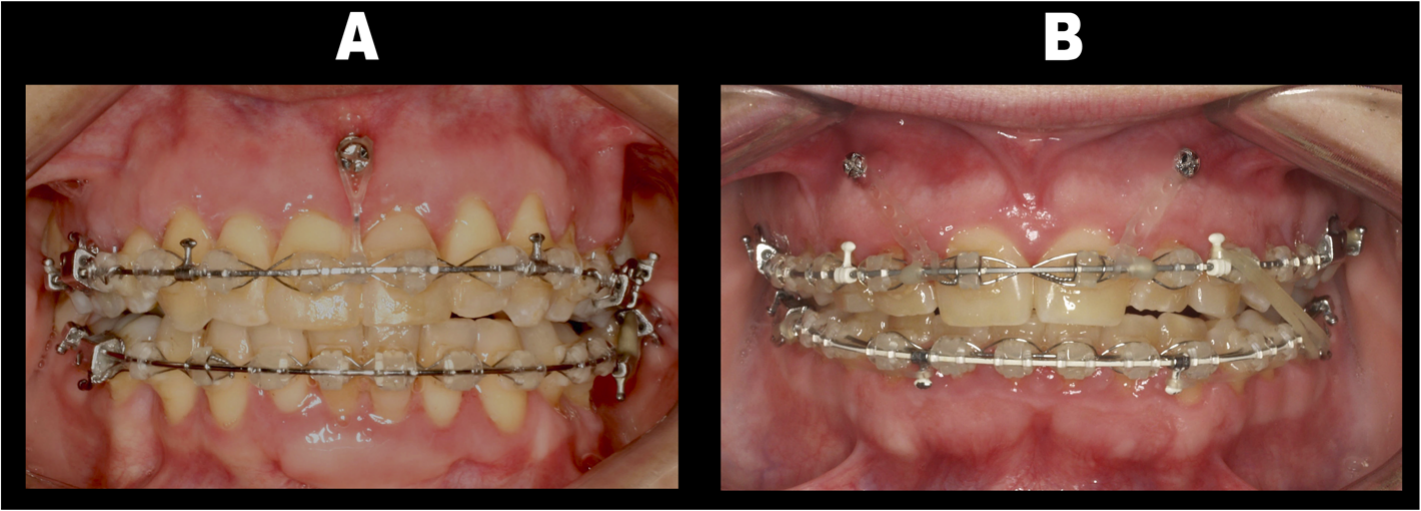
Miniscrew positions in the two groups. **a** One miniscrew placed between upper central incisors. **b** Two miniscrews placed between upper lateral incisors and canines

**Fig. 2:**
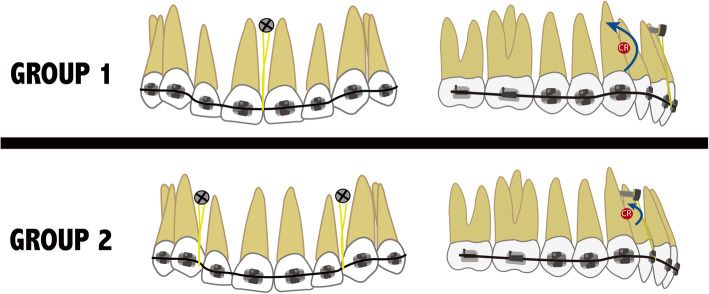
Illustrations of the mechanics used for upper incisor intrusion and their effects in group 1 (one miniscrew) and group 2 (two miniscrews)

Patients were divided into two groups depending on the number of miniscrews placed and their location (Fig. 1). The decision of whether to place one miniscrew or two depended on the root inclination and position of the labial frenum. In group 1 (Figs. 1a and 2), a single miniscrew was placed in the upper incisor area between the upper central incisors, located anterior to the center of resistance (CR), aiming to achieve less intrusion and more labial tipping of the incisors. In group 2 (Figs. 1b and 2), two miniscrews were placed between upper lateral incisors and canines. Since both miniscrews were placed more posterior (being the distance to CR shorter), more intrusion with less labial tipping was expected [13].

### Cephalometric analysis

Three lateral cephalometric radiographs were obtained for each patient: before treatment (T0), after treatment (T1), and during the retention period (T2).

**Fig. 3:**
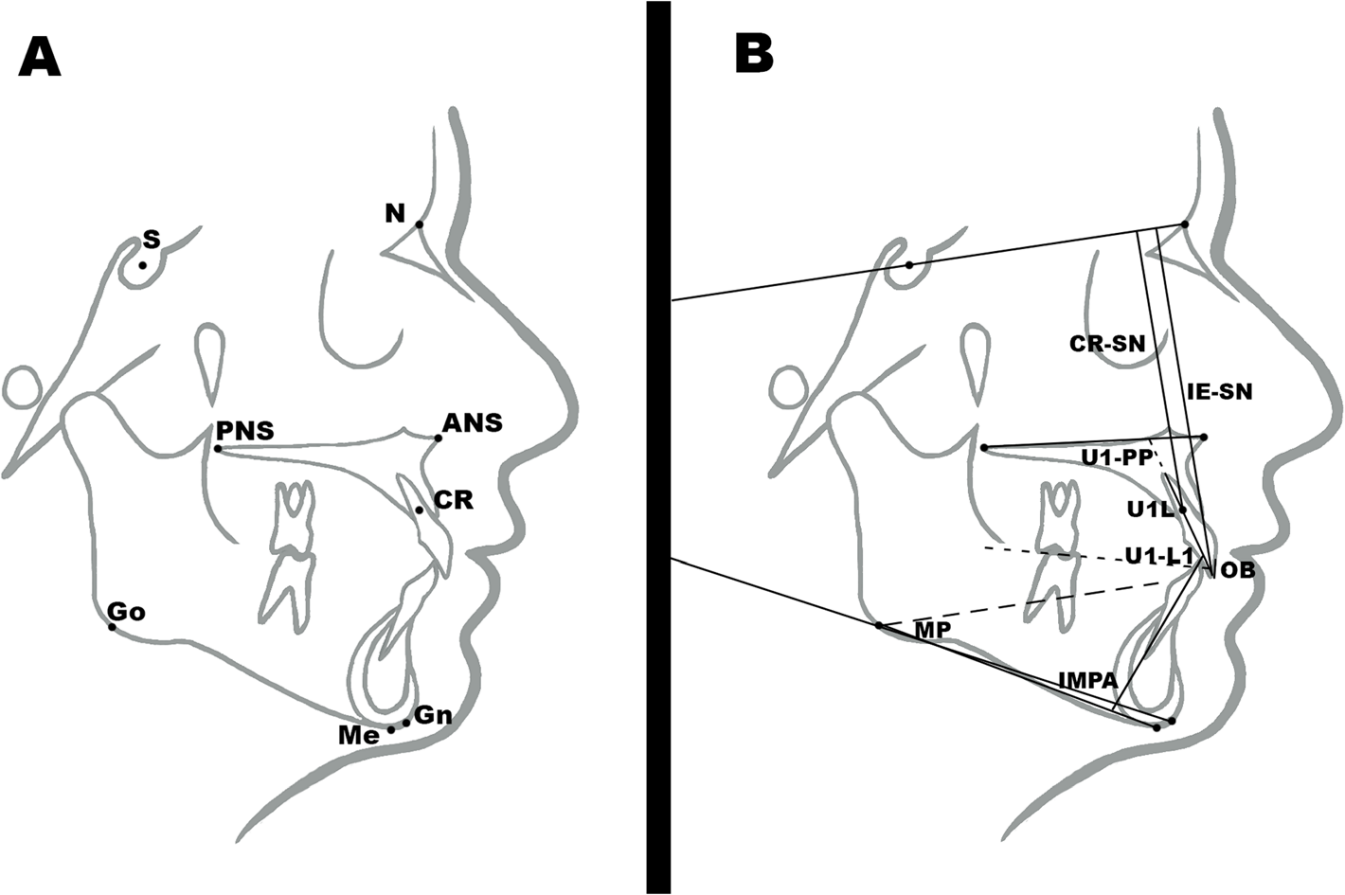
**a** Cephalometric landmarks used in the study described in Table 1. **b** Cephalometric skeletal and dental measurements used in the study described in Table 2

Eight cephalometric landmarks were identified on each radiograph: S, N, Gn, Go, Me, ANS, PNS, and CR (Fig. 3a) and eight skeletal and dental measurements were taken (Table 1 and Fig. 3b) by a single observer who had been fully trained and calibrated (LGZ). The same set of measurements were repeated by a second calibrated observer (FLL). All cephalometric measurements were taken using Nemoceph® 11.3.1 software.

**Table 1:** Cephalometric measurements (8) abbreviations and definitions used in the study. Four linear and 4 angular measurements

Cephalometric measurement		Abbreviation	Definition
**1**	Mandibular plane angle (°) (Steiner)	MP	Angular measurement. Angle formed by the intersection of Mandibular plane (Go-Gn) and SN. Norm 32° ± 4°
**2**	Overbite (mm)	OB	Linear measurement. Distance in millimeters between upper and lower incisal edges perpendicular to the occlusal plane. Norm 2 mm
**3**	Lower incisor inclination (°) (Tweed)	IMPA	Angular measurement. Angle formed by the intersection of lower incisor (L1) and mandibular plane (Me-Go). Norm 90° ± 2.5°
**4**	Upper incisor inclination (°) (Burstone)	U1-PP	Angular measurement. Angle formed by the intersection of upper incisor (U1) and palatine plane (ANS-PNS). Norm 112°–117°
**5**	Upper Incisor Length (mm)	U1L	Linear measurement. Distance in millimeters between the upper incisor incisal edge and root apex. No norm
**6**	Upper incisor position 1 (mm)	CR-SN	Linear measurement. Perpendicular distance in millimeters between the upper incisor center of resistance and SN. No norm
**7**	Upper incisor position 2 (mm)	IE-SN	Linear measurement. Perpendicular distance in millimeters between the upper incisal edge and SN. No norm
**8**	Interincisal angle (°)	U1-L1	Angular measurement. Angle formed by the intersection of upper incisor (U1) and lower incisor (L1). Norm 130°

For each patient, radiographs taken at T0, T1, and T2 were superimposed to (1) assess the changes produced by orthodontic treatment (T1–T0); (2) assess changes that had taken place during the retention period (T2–T1).

### Outcomes

The following parameters were evaluated:

- Results of orthodontic movement by one or two anterior miniscrews for upper incisor correction of overbite and angulation in adult patients.
- Upper incisor resorption deriving from 1 or 2 miniscrews and their locations.
- The stability of both treatment options.

### Statistical analysis

Power analysis showed that a sample size of at least 40 patients would provide an 80% probability of detecting a medium effect (*f* = 0.2) between time-points, using an ANOVA model at a confidence level of 95%, and assuming a correlation among repeated measurements of 0.5.

Intraobserver and interobserver error was calculated by coefficients of variation (CV = SD × 100/mean, expressed as percentages) and by the Dahlberg formula. All lateral radiographs (132) were traced and measured again one week later by the principal observer (LGZ) and by a second calibrated observer (FLL).

Data obtained from cephalometric measurements were entered on a spreadsheet, using the Microsoft® Excel 2011® program. Study variables were the dental measurements (both lineal and angular) taken at T0, T1, and T2. Descriptive statistics were calculated for each parameter, as well as the differences between times (T1–T0; T2–T1; T2–T0). Differences between times represented the effect of treatment (T1–T0), stability (T2–T1), and long-term overall effect (T2–T0). The normality of the measurement differences was checked by the Kolmogorov-Smirnov test, obtaining a confirmatory result (*p* > 0.05) for all parameters. A linear model repeated measures ANOVA was used to evaluate the effects of treatment at different times. Pearson’s correlation coefficient was applied to evaluate different parameters between T1 and T0. The level of significance established was 5% (*p* = 0.05).

## Results

After applying inclusion and exclusion criteria, 46 patients were included in the study. Two patients in group 1 were excluded due to miniscrew loosening, so the final patient sample consisted of 44 patients, 24 (54.54%) women, and 20 (45.45%) men; this being a homogeneous distribution. Mean patient age was 36.6 ± 4.9 years. Ten female and six male patients with a mean age of 35.6 ± 6.3 years comprised the group with one miniscrew, while the group treated with two miniscrews was made up of 14 females and 14 males subjects with a mean age of 34.6 ± 3.48. Mean total treatment duration was 23.3 ± 7.7 months, and miniscrews were used for a mean period of 6.1 months ± 1.2. After the intrusion period, the miniscrews were kept in the mouth for a couple of months with a stainless steel ligature. The mean orthodontic retention period after treatment was 31.1 months ± 7.1.

Intra- and inter-observer error was appropriate: *d* of Dahlberg was under 0.28 and CVs were below 2.55% in all cases.

The measurements taken at the three evaluation times (T0, T1, and T2) are shown in Table 2; ANOVA analysis was used to determine whether differences between times were statistically significant (Table 3). Firstly, measurements from all patients were assessed together without separating the groups (one or two miniscrews). Secondly, measurements taken at the three times by group (one or two miniscrews). All values except for the mandibular plane underwent statistically significant changes as a result of treatment. Differences in cephalometric measurements between groups for T0, T1, and T2 are shown in Table 4.

**Table 2:** Cephalometric measurements for T0, T1, and T2: mean ± standard deviation (SD), minimum/maximum and median

M Measurements	Group	T0			T1			T2		
		Mean ± SD	Min/max	Median	Mean ± SD	Min/max	Median	Mean ± SD	Min/max	Median
**U1L (mm)**	Overall	21.57 ± 2.07	17.76/25.60	21.30	19.42 ± 2.05	14.92/23.00	18.92	19.40 ± 2.04	14.99/23.00	18.89
	1	21.61 ± 1.69	19.18/25.09	21.64	19.41 ± 2.00	14.92/22.98	18.90	19.39 ± 2.01	14.97/22.90	18.87
	2	21.54 ± 2.34	17.76/25.60	21.09	19.43 ± 2.04	14.93/23.00	19.02	19.40 ± 1.99	14.97/22.93	18.84
**OB (mm)**	Overall	5.59 ± 2.21	1.80/12.10	5.10	2.36 ± 1.68	− 0.55/6.53	2.25	2.45 ± 1.62	− 0.25/6.93	2.45
	1	5.17 ± 2.47	1.80/12.10	4.65	2.42 ± 1.93	− 0.55/6.53	2.25	2.50 ± 1.62	− 0.25/6.93	2.45
	2	6.20 ± 1.50	3.70/7.90	6.86	2.40 ± 1.68	− 0.55/6.53	2.25	2.48 ± 1.62	− 0.25/6.93	2.45
**MP (°)**	Overall	33.87 ± 6.15	19.68/45.28	35.08	33.9 ± 6.94	17.68/46.28	35.67	34.06 ± 6.75	16.68/46.28	35.32
	1	30.33 ± 5.32	21.16/38.04	32.42	30.03 ± 6.13	19.16/36.00	31.55	30.45 ± 5.45	20.16/37.00	31.02
	2	35.16 ± 6.50	19.68/45.28	36.30	35.77 ± 7.03	17.68/46.28	37.46	35.75 ± 7.11	16.68/46.28	37.30
**CR-SN (mm)**	Overall	71.50 ± 5.18	63.45/82.62	71.06	67.66 ± 4.58	57.96/78.35	67.91	67.72 ± 4.66	58.00/78.37	67.86
	1	77.47 ± 4.29	71.82/82.62	78.36	71.78 ± 3.99	67.23/78.35	70.64	71.92 ± 4.09	67.22/78.37	70.59
	2	74.60 ± 3.49	63.45/75.59	69.45	66.41 ± 3.73	57.96/73.83	66.62	66.44 ± 3.76	58.00/74.00	66.69
**IE-SN (mm)**	Overall	81.34 ± 4.93	73.46/93.03	80.93	77.36 ± 4.51	69.55/88.70	77.48	77.53 ± 4.39	69.74/88.70	77.61
	1	86.89 ± 3.81	81.85/93.03	86.91	81.31 ± 4.06	77.51/88.70	79.76	81.38 ± 4.26	77.05/88.70	79.67
	2	84.51 ± 3.65	73.46/86.89	79.76	76.13 ± 3.72	69.55/84.44	76.17	76.22 ± 3.62	69.74/84.16	75.97
**U1-PP (°)**	Overall	95.23 ± 9.25	78.20/115.00	97.00	106.82 ± 5.81	95.16/116.44	107.66	106.36 ± 6.18	94.36/119.44	107.06
	1	95.14 ± 12.28	78.20/115.0	100.4	109.44 ± 4.81	99.00/116.00	109.94	108.81 ± 4.91	98.00/115.00	109.50
	2	94.50 ± 6.98	79.00/104.12	96.24	106.08 ± 6.17	95.16/116.44	106.52	105.78 ± 6.75	94.36/119.44	105.52
**IMPA (°)**	Overall	88.99 ± 7.88	75.16/103.92	88.96	97.69 ± 9.31	79.16/115.00	98.27	97.25 ± 9.16	79.88/115.00	99.00
	1	89.42 ± 9.69	77.00/103.92	89.14	98.05 ± 10.49	80.20/112.92	98.94	97.07 ± 10.03	81.20/111.92	97.94
	2	89.75 ± 7.09	75.16/101.00	90.73	98.04 ± 9.39	79.16/115.00	99.27	98.02 ± 9.16	79.88/115.00	99.86
**U1-L1 (°)**	Overall	145.62 ± 14.01	122.04/174.80	143.60	126.32 ± 8.45	103.52/145.60	127.63	126.35 ± 8.10	104.52/143.60	127.68
	1	142.09 ± 9.77	131.30/169.00	143.04	125.77 ± 7.97	103.52/136.00	126.10	125.95 ± 9.28	115.08/143.60	123.70
	2	144.61 ± 9.77	130.10/165.20	144.05	126.87 ± 7.77	104.22/135.01	127.09	125.68 ± 7.85	104.52/134.72	127.02

*U1L* upper incisor length, *OB* overbite, *MP* mandibular plane, *CR*-*SN* upper incisor position 1, *IE-SN* upper incisor position 2, *U1-PP* upper incisor inclination, *IMPA* lower incisor inclination, *U1-L1* interincisal angle

**Table 3:** Differences in means of cephalometric measurements at T0 (before treatment), T1 (after treatment), and T2 (during retention): mean ± standard deviation (SD), minimum/maximum, and median. Post hoc test with Bonferroni correction ANOVA model of repeated measurements (*p* values for changes over time and homogeneity of changes between groups or interaction)

Measurements	Group	T1–T0					T2–T1					T2–T0				
		Mean ± SD	Min/max	Median	***p***	***p*** (int)	Mean ± SD	Min/max	Median	***p***	***p*** (int)	Mean ± SD	Min/max	Median	p	p (int)
**U1L (mm)**	Overall	− 2.15 ± 0.85 *	− 3.00/− 1.01	− 2.09	< 0.001*		− 0.02 ± 0.04	− 0.13/0.05	0.00	0.162		− 2.17 ± 0.85 *	− 3.02/− 1.02	− 2.10	< 0.001*	
	1	− 2.20 ± 0.88 *	− 3.00/− 0.99	− 2.13	< 0.001*	0.779	− 0.02 ± 0.04	− 0.11/0.05	0.00	0.163	1.000	− 2.22 ± 0.89 *	− 3.02/− 0.99	− 2.10	< 0.001*	0.843
	2	− 2.11 ± 0.82 *	− 2.88/− 1.01	− 2.08	< 0.001*		− 0.03 ± 0.04	− 0.13/0.04	0.00	0.155		− 2.14 ± 0.82*	− 2.98/− 1.02	− 2.04	< 0.001*	
**OB (mm)**	Overall	− 3.23 ± 1.73 *	− 6.90/− 0.20	− 3.00	< 0.001*		0.09 ± 0.29	− 0.60/0.60	0.10	0.167		− 3.15 ± 1.81*	− 6.80/− 0.30	− 2.95	< 0.001*	
	1	− 2.75 ± 1.63 *	− 6.90/− 0.17	− 2.55	<0.001*	0.074	0.08 ± 0.28	− 0.57/0.60	0.10	0.162	0.889	− 2.67 ± 1.76*	− 6.80/− 0.25	− 2.95	0.035*	0.044*
	2	− 3.80 ± 1.43 *	− 6.55/− 0.20	− 3.77	< 0.001*		0.08 ± 0.27	− 0.60/0.55	0.08	0.170		− 3.72 ± 1.86*	− 6.50/− 0.30	− 3.47	< 0.001*	
**MP (°)**	Overall	0.04 ± 2.05	− 5.00/3.20	0.60	1.000		0.15 ± 1.11	− 3.00/2.00	0.00	1.000		0.19 ± 1.84	− 3.00/3.00	0.50	1.000	
	1	− 0.30 ± 1.95	− 5.00/3.10	0.50	1.000	1.000	0.42 ± 0.18	− 2.94/2.00	− 0.02	1.000	1.000	0.12 ± 1.54	− 3.00/2.89	0.32	1.000	1.000
	2	0.61 ± 2.15	− 4.92/3.20	0.71	1.000		− 0.02 ± 0.08	− 3.00/1.96	0.02	1.000		0.59 ± 1.99	− 2.91/3.00	0.55	1.000	
**CR-SN (mm)**	Overall	− 3.84 ± 2.96 *	− 12.4/− 0.58	− 3.45	< 0.001*		0.06 ± 0.29	− 0.49/0.95	− 0.02	0.640		− 3.79 ± 2.98 (*)	− 12.4/− 0.54	− 3.60	< 0.001*	
	1	− 5.69 ± 2.66 *	− 12.4/− 0.52	− 4.45	< 0.001*	0.041*	0.14 ± 0.29	− 0.46/0.95	− 0.02	0.635	0.931	− 5.55 ± 2.78*	− 12.4/− 0.50	− 4.60	0.029*	0.033*
	2	− 8.19 ± 3.66 *	− 11.8/− 0.58	− 7.25	< 0.001*		0.03 ± 0.17	− 0.49/0.93	0.02	0.712		− 8.16 ± 3.98*	− 11.9/− 0.54	− 7.29	< 0.001*	
**IE-SN (mm)**	Overall	− 3.98 ± 2.81 *	− 11.3/− 0.71	− 3.67	< 0.001*		0.17 ± 0.54	− 0.54/1.96	0.08	0.127		− 3.81 ± 2.84 (*)	− 11.1/− 0.47	− 3.74	< 0.001*	
	1	− 5.58 ± 2.51 *	− 11.3/− 0.66	− 4.67	< 0.001*	0.032*	0.07 ± 0.43	− 0.51/1.96	0.08	0.227	1.000	− 5.51 ± 2.64 *	− 11.1/− 0.41	− 4.74	0.042*	0.027*
	2	− 8.38 ± 2.99 *	− 10.7/− 0.71	− 6.91	< 0.001*		0.09 ± 0.64	− 0.54/1.92	− 0.07	0.321		− 8.29 ± 3.05*	− 10.8/− 0.47	− 6.01	< 0.001*	
**U1-PP (°)**	Overall	11.6 ± 9.03 *	− 5.00/30.8	10.3	< 0.001*		− 0.47 ± 1.85	− 2.40/6.00	− 1.00	0.304		11.1 ± 9.78(*)	− 5.00/33.0	8.95	< 0.001*	
	1	14.3 ± 9.99 *	− 5.00/28.9	13.3	< 0.001*	0.048*	− 0.63 ± 1.97	− 2.37/6.00	− 0.95	0.299	0.852	13.7 ± 9.48*	− 5.00/32.0	12.9	< 0.001*	0.038*
	2	11.58 ± 8.03*	− 4.88/30.8	10.99	< 0.001*		− 0.30 ± 1.55	− 2.40/5.96	− 1.10	0.331		11.3 ± 9.99 *	− 4.77/33.0	10.0	0.042*	
**IMPA (°)**	Overall	8.70 ± 8.51 *	− 4.00/35.0	7.70	< 0.001*		− 0.44 ± 1.86	− 3.00/5.00	− 0.45	0.368		8.26 ± 8.10(*)	− 0.50/35.0	7.50	0.001*	
	1	8.63 ± 8.71 *	− 4.00/34.0	7.70	< 0.001*	1.000	− 0.98 ± 1.96	− 2.96/5.00	− 0.47	0.388	1.000	7.65 ± 7.70*	− 0.50/33.6	7.50	0.001*	1.000
	2	8.29 ± 8.31 *	− 3.91/35.0	7.32	< 0.001*		− 0.02 ± 1.71	− 3.00/4.59	− 0.39	0.393		8.27 ± 8.54*	− 0.46/35.0	7.12	0.002*	
**U1-L1 (°)**	Overall	− 19.3 ± 15.3 *	− 63.0/− 1.00	− 14.0	< 0.001*		0.03 ± 1.49	− 4.00/2.30	0.00	1.000		− 19.3 ± 15.5(*)	− 62.0/0.00	− 14.0	< 0.001*	
	1	− 16.3 ± 14.3 *	− 63.0/− 0.95	− 14.0	< 0.001*	0.702	0.18 ± 1.59	− 3.91/2.30	0.00	1.000	1.000	− 16.1 ± 14.5 *	− 62.0/− 0.04	− 14.0	< 0.001*	0.692
	2	− 18.8 ± 17.4 *	− 62.4/− 1.00	− 16.2	< 0.001*		− 0.09 ± 1.34	− 4.00/2.27	0.00	1.000		− 18.9 ± 16.7*	− 61.5/0.00	− 15.9	< 0.001*	

*Statistically significant difference (*p* < 0.001)

*U1L* upper incisor length, *OB* overbite, *MP* mandibular plane, *CR-SN* upper incisor position 1, *IE-SN* upper incisor position 2, *U1-PP* upper incisor inclination, *IMPA* lower incisor inclination, *U1-L1* interincisal angle

**Table 4:** Differences of cephalometric measurements for T0, T1, and T2 between groups: mean ± standard error (SE). Post hoc test with Bonferroni correction ANOVA model of repeated measurements (*p* values)

M Measurements	T0		T1		T2	
	Mean ± SE	***p*** value	Mean ± SE	***p*** value	Mean ± SE	***p*** value
**U1L (mm)**	0.07 ± 0.67	1.000	− 0.02 ± 0.63	1.000	− 0.01 ± 0.63	1.000
**OB (mm)**	− 1.03 ± 0.60	0.286	0.02 ± 0.56	1.000	0.02 ± 0.51	1.000
**MP (°)**	− 4.83 ± 1.91	0.053	− 5.74 ± 2.11	0.033*	− 5.30 ± 2.06	0.047*
**CR-SN (mm)**	2.87 ± 1.19	0.068	5.37 ± 1.20	0.001*	5.16± 1.21	0.001*
**IE-SN (mm)**	2.38 ± 1.16	0.150	5.18 ± 1.20	0.001*	5.16 ± 1.21	0.001*
**U1-PP (°)**	0.64 ± 2.89	1.000	3.36 ± 1.79	0.114	3.03 ± 1.93	0.183
**IMPA (°)**	− 0.33 ± 2.54	1.000	0.01 ± 3.07	1.000	− 0.95 ± 2.97	1.000
**U1-L1 (°)**	− 2.52 ± 3.06	1.000	− 1.10 ± 2.46	1.000	0.27 ± 2.63	1.000

*U1L* upper incisor length, *OB* overbite, *MP* mandibular plane, *CR-SN* upper incisor position 1, *IE-SN* upper incisor position 2, *U1-PP* upper incisor inclination, *IMPA* lower incisor inclination, *U1-L1* interincisal angle

Upper incisor resorption after treatment was 2.15 ± 0.85 mm (9.9% of the initial length of the tooth), being 2.20 ± 0.88 for group 1 and 2.11 ± 0.82 for group 2. Tooth length remained stable after treatment. Pearson’s correlation coefficient was used to determine whether upper incisor resorption was related to variations in other parameters. Table 5 shows the correlation coefficients and statistical significance for each pair. Upper incisor resorption was significantly related to overbite correction. A simple linear regression model was used to assess this correlation and a beta coefficient value of 0.193 ± 0.051 was obtained, meaning that for each millimeter of overbite reduction, 0.19 mm of root resorption was produced. The ANOVA model concluded that there were no statistically significant differences in root resorption between the two groups.

**Table 5:** Correlation between upper incisor resorption and other parameter variations between T1–T0

Upper incisor resorption	T1–T0
**Treatment duration**	*r* = 0.22; *p* = 0.145
**OB**	*r* = 0.43 ; *p* = 0.004**
**MP**	*r* = − 0.14; *p* = 0.356
**CR-SN**	*r* = 0.10; *p* = 0.520
**IE-SN**	*r* = 0.09; *p* = 0.570
**U1-PP**	*r* = − 0.27; *p* = 0.071
**IMPA**	*r* = − 0.18; *p* = 0.249
**U1-L1**	*r* = 0.24 ; *p* = 0.125

*p < 0.05

**p < 0.01

***p < 0.001

*OB* overbite, *MP* mandibular plane, *CR-SN* upper incisor position 1, *IE-SN* upper incisor position 2, *U1-PP* upper incisor inclination, *IMPA* lower incisor inclination, *U1-L1* interincisal angle

Mean overbite decrease was − 3.23 ± 1.73 mm and relapsed by just 0.09 ± 0.29 mm. Overbite correction was achieved by upper incisor intrusion and lower incisor inclination but no counterclockwise rotation of the mandible was produced, since the mandibular plane angle did not undergo any statistically significant change as a result of treatment. The ANOVA model concluded that there was more overbite reduction in the group treated with two miniscrews located between the lateral incisors and canines (− 3.80 ± 1.43 versus − 2.75 ± 1.63). Upper incisor intrusion was observed in all patients indicated by the two measurements CR-SN and IE-SN; these did not undergo any significant relapse. Intrusion was greater in group 2 (two miniscrews), being this difference statistically significant. Regarding upper incisor angulation (U1-PP), an increase was achieved with no statistically significant relapse. Unlike intrusion, angulation was greater in group 1 (14.3 ± 9.99) than in group 2 (11.58 ± 8.03) with statistically significant difference (*p* = 0.048). However, less incisor angulation (IMPA) increase was observed with no statistically significant relapse. Unlike upper incisor angulation, no significant differences between groups were found. Due to variations in upper and lower incisor angulation, interincisal angle underwent a significant decrease with no relapse, although no significant differences between groups were found.

## Discussion

Incisor intrusion assisted by miniscrews has gained popularity in recent years, as miniscrews reduce the need for complicated mechanics and avoid the side effects of more conventional methods [19]. The present study analyzed the changes produced during miniscrew-assisted orthodontic treatment focusing on intrusion pattern, while the other factors assessed were a consequence of this intrusion. As shown in the present study, deep overbite can be corrected within a short period of time. Understanding the mechanisms, cephalometric changes and adverse effects related to overbite reduction using different treatment approaches can help clinicians make treatment planning more precise.

In the present study, patients presenting maxillary incisors with a history of some kind of trauma, endodontic treatment, or patients with any systemic disease or periodic medication were excluded since there is a relationship between these disorders and root resorption [24–27].

The study showed that significant changes occurred as a consequence of orthodontic treatment assisted by miniscrews. It should be noted that these changes were a result of the combination of intrusion by vertical force from miniscrews and the effects of bracket wires.

With regard to the present study’s outcomes, the mandibular plane was the only result that did not undergo significant changes as a result of treatment. No counterclockwise rotation of the mandible was produced by this type of treatment, which concurs with the findings of previous research [12]. This proves that deep overbite correction by means of miniscrews produces more genuine incisor intrusion and less molar extrusion, and so does not produce significant counterclockwise rotation of the mandibular plane [11, 28].

All the measurements were taken from lateral cephalograms, following the methodology established in most other studies of similar design [28]. Although a few authors have measured root resorption from CBCTs [14, 19], this being a more accurate method, we did not consider taking CBCT scans justifiable in the context of the present study.

Vertical incisor movement was measured using two different reference points (incisal edge and CR), making it possible to compare the results with a wider range of studies. Several authors [15] have used these two landmarks to assess incisor intrusion. The CR was set as 40% of the distance from the alveolar crest to the root apex [29]. The CR is a more reliable point since it is not affected by incisor inclination, unlike the incisal edge or root apex [30]. Unlike studies that have used the palatal plane as reference for these measurements (ANS-PNS) [12, 14], the present study used the SN plane, as it is considered more reliable for studies of intrusion since the palatal plane has been shown to move slightly after intrusion [11].

Patients were allocated to one of two groups depending on root inclination and frenum. In group 1, one miniscrew was placed in the interradicular space between the two central incisors, this location being anterior to the CR. In this way, the force applied produced less intrusion but more buccal tipping. In group 2, two miniscrews were inserted between the roots of canines and lateral incisors. In this way, force was applied more posteriorly but still anterior to the CR, producing less labial tipping but more intrusion. These effects have already been described by Lindauer and Isaacson [13], who demonstrated that the different effects that obtained during intrusion and extrusion movements depend on the point where force is applied in relation to the CR of the anterior teeth. Although buccal tipping produced by miniscrew mechanics could be considered an undesirable effect, this is often not the case as many of the patients presenting overbite and gummy smile may present retroclination of the upper incisors, making buccal inclination a favorable effect leading to better and more stable outcomes. It should be noted that in group 2, the total force applied from the miniscrews was greater than that applied in group 1 (180 g and 90 g respectively), which could alter the velocity of movement and the amount of root resorption.

Since no comparative clinical studies on the effects of miniscrews in relation to the incisor area where they are inserted have been published, one of the aims of the present study was to assess the overall root resorption produced by incisor intrusion when using miniscrews, and to analyze the differences in root resorption between one and two miniscrews located in different areas. Our results showed that overall root resorption was 2.15 ± 0.85 mm with no statistically significant differences between the two groups. Other studies of incisor intrusion have obtained lower root resorption values when using miniscrews [7, 19, 31] or conventional intrusion archwires [14, 32, 33]. These differences may be due to the amount of intrusion produced, as there is a positive correlation between intrusion and resorption rates, as the present study demonstrates, the amount of intrusion found in the present study being higher than amounts reported in other studies (3.84 ± 2.96 mm). Dermaut et al. [34] found higher resorption rates (2.5 mm) when using the Burstone intrusion technique.

Some authors have found that lingual root torque was a strong predictor of external root resorption [35]. In this regard, our results show significantly greater incisor buccal inclination in group 1, root resorption also being higher in this group. It should be noted that several additional patient-based factors can affect root resorption rates, such as a long and narrow root shape, deviated root, or proximity to the cortical plates [27].

Intrusion values were found to be higher in the present study than those reported by other authors using conventional methods, such as utility arches or Burstone intrusion arches [7, 32–34, 36, 37]. Our results show that the amount of upper incisor resorption depends on the amount of intrusion, the results being in agreement with other studies [20–22, 34] even though the methods used by other authors were different to those in the present study: intrusive forces applied to premolars rather than incisors, or forces applied by means of appliances other than miniscrews, or forces applied directly to teeth rather than to archwires.

Although differences between groups were found for all the factors analyzed, most of them did not show statistical significance despite the major differences in force vectors. This fact may be due to other factors affecting orthodontic movement, such as the level of crowding present or archwire effects.

The results of the present study showed that the use of miniscrews for incisor intrusion provided good stability for all measurements in both groups. But the stability results cannot be compared to any other studies since none of the published works on incisor intrusion with miniscrews have reported this data, as noted in the single systematic review conducted to date [28].

Although resorption occurred in all teeth, the degree of root resorption recorded can be considered clinically irrelevant and in any case ceased when treatment came to an end. Besides, when resorption percentages were considered, length losses were relatively small.

This study suffered several limitations. Firstly, a two-dimensional method was used to measure root resorption but, as resorption constitutes a volume loss, a three-dimensional quantitative method such as CBCT would be much more precise [19]. However, the patients did not have CBCTs and taking CBCTs just for the purposes of the study was not considered justifiable. Secondly, lateral incisor root resorption was not considered, although some authors have found no differences in resorption between lateral and central incisors [34]. Thirdly, variations in the type (continuous or transient) and magnitude of force, duration of intrusion, and measurement methods using conventional radiographs made it difficult to compare the present results with previous studies. Lastly, the groups could not be randomized since the allocation was based on the position of the roots and labial frenum.

## Conclusions

According to the results of the present study, it may be concluded that:

-Overbite correction may be achieved successfully by a combination of upper incisor intrusion and lower incisor proclination with no rotation of the mandibular plane using one or two miniscrews. Upper incisor buccal angulation increase is greater in patients treated with one miniscrew, while upper incisor intrusion and overbite correction are greater in patients treated with two.
-Root resorption is slightly over 2 mm, being positively related to the amount of intrusion with no significant differences between cases treated with one or two miniscrews; it ceases at the end of active treatment.
-Stability is satisfactory when using either one or two miniscrews.

## Data Availability

The datasets used and/or analyzed in the course of this study are available from the corresponding author on reasonable request.

## Abbreviations

ANOVA: Analysis of variance
CBCT: Cone beam computed tomography
STROBE: Strengthening the Reporting of Observational Studies in Epidemiology
SE-NT: Superelastic nickel-titanium
CR: Center of resistance
CV: Coefficient of variation
SD: Standard deviation
